# Parent–Child Interaction Therapy for Disruptive Behavior: A Systematic Review of Effectiveness in Different Settings

**DOI:** 10.3390/jcm14030856

**Published:** 2025-01-28

**Authors:** Andrea Calderone, Adriana Piccolo, Desirèe Latella, Rosaria De Luca, Francesco Corallo, Angelo Quartarone, Angela Militi, Francesca Cucinotta, Rocco Salvatore Calabrò

**Affiliations:** 1 Department of Clinical and Experimental Medicine, University of Messina, 98100 Messina, Italy; andrea.calderone95@gmail.com; 2 IRCCS Centro Neurolesi Bonino Pulejo, 98124 Messina, Italy; desiree.latella@irccsme.it (D.L.); rosaria.deluca@irccsme.it (R.D.L.); francesco.corallo@irccsme.it (F.C.); angelo.quartarone@irccsme.it (A.Q.); francesca.cucinotta@irccsme.it (F.C.); roccos.calabro@irccsme.it (R.S.C.); 3 Department of Biomedical, Dental Science and Morphological and Functional Images, University of Messina, 98100 Messina, Italy; angela.militi@unime.it

**Keywords:** parent–child interaction therapy, disruptive behavior, rehabilitation

## Abstract

**Background:** Parent–child interaction therapy (PCIT) is an evidence-based intervention for children with behavioral problems. This systematic review assesses the efficacy of PCIT in reducing disruptive behavior problems (DBPs) by analyzing outcomes across diverse populations and settings. **Methods:** Studies were identified through an online search of the PubMed, Web of Science, Cochrane Library, and Embase databases, with a search period ranging from 2014 to 2024. This review was registered on Open OSF (n) TVFQ5. **Results:** Research studies demonstrate that PCIT is effective across a variety of DBP conditions, showing significant reductions in child behavioral problems and parenting stress. PCIT’s adaptability, effectiveness, and potential for widespread use have been validated in both specialized and community settings, including diverse and high-risk populations. **Conclusions:** This systematic review highlights PCIT’s overall effectiveness in reducing disruptive behaviors and improving parent–child relationships across diverse settings and populations. It emphasizes the need for further research into its long-term efficacy, adaptability in multicultural contexts, and potential integration with technology for enhanced dissemination and effectiveness.

## 1. Introduction

Disruptive behavior problems (DBPs) are highly prevalent among children, affecting an estimated 10–15% of the population [1]. DBPs often include displays of anger and irritability, opposition, defiance, and aggression [2], and they constitute an important public health challenge, with significant impacts on conduct behaviors and an increased risk of developing mental health complications in adulthood [3,4,5]. Although such behaviors are sometimes considered a normal phase of child development, their frequency and intensity can vary and lead to significant consequences if not addressed appropriately [6]. In some cases, it tends to resolve spontaneously and can be interpreted as a normal response to the developmental challenges of early childhood, rather than as signs of pathology [7]. However, approximately 50% of these children continue to exhibit these behaviors, which may represent the beginning of a trajectory toward externalizing problems and conduct disorders during childhood, with negative consequences at the socio-emotional, academic, and social levels [8,9]. The onset of these behavioral problems can stem from various causes, often originating from biological, psychological, environmental, and social factors [10]. Despite their differing etiological origins, these behaviors are present in all living environments and have a great impact on children and families [11,12]. A specific intervention has been shown to be effective in managing DBPs when implemented in an ecological and generalized manner [13]. In this context, multidisciplinary interventions aim to involve parents, teachers, schools, and community support to teach discipline techniques [14], increase parental warmth, and enhance parent–child relationships [15]. Among the range of treatment options available, Parent–Child Interaction Therapy (PCIT) has emerged as a particularly promising approach, especially in addressing the relationship dynamics between children and their caregivers. PCIT, designed by Dr. Sheila Eyberg, combines principles from both cognitive behavioral therapy and operant conditioning for children aged 2–7 years with Disruptive Behavior Disorder (DBD) [16]. There are two key components of PCIT: child-directed interaction and parent-directed interaction. The former strengthens the relationship between the child and the parent and leads to more positive interactions. Through this process, parents learn skills related to praise, reflection, and imitation of their child’s behavior, thereby increasing the child’s self-esteem and willingness to cooperate [17]. A sense of appreciation and being heard is conveyed to a child within a nurturing environment. The second aspect of therapy addresses behavioral maladjustments through parent training in effective disciplinary processes. This principle is designed to train parents in the appropriate and correct use of clear and consistent commands, along with expectations and appropriate consequences for misconduct. It emphasizes techniques or procedures, such as time-out, privilege revocation, and consistency in rule enforcement. It should, therefore, focus on training parents to effectively manage the child’s behavior and ways to create an orderly environment that fosters self-regulation and compliance [18,19]. PCIT is implemented with the use of live coaching. Here, the therapist observes live interactions between the parent and the child behind a one-way mirror, while the parent receives live feedback through a radio earpiece [20,21]. In this way, immediate guidance can be given on the parents’ conduct to properly implement the procedures; thus, the strategies are implemented effectively. Again, the therapist helps to overcome specific barriers and models the application of skills in everyday situations [22,23]. Extensive research underscores the effectiveness of PCIT in managing disruptive behaviors across various clinical contexts. Vetter (2018) demonstrated that PCIT significantly improved parent–child interactions and reduced behavioral problems in children with autism spectrum disorder (ASD) and attention deficit/hyperactivity disorder (ADHD), highlighting its adaptability to complex behavioral issues [24]. Similarly, Cambric and Agazzi (2019) reported notable improvements in behavior and compliance in a case study involving a child with high-functioning ASD, emphasizing its potential in addressing specific challenges faced by children in mainstream settings [25]. Additionally, a review by Rayfield et al. confirmed PCIT’s efficacy in reducing oppositional and defiant behaviors while enhancing parent–child relationships, further validating its role in treating disruptive behaviors [26]. In the context of early childhood, Setiyowati et al. found that PCIT effectively reduced the frequency and severity of tantrums in preschool children, positioning it as a key intervention for affect regulation in early development [27].

Although PCIT has been frequently studied for the treatment of aggressive behaviors in DBD, its application can also be useful in other clinical and psychosocial settings. In this sense, the present study aimed to critically analyze the existing literature on the application of PCIT in different contexts where aggressive behaviors can manifest as secondary symptoms or in association with other diagnostic pictures. Therefore, the aim of this review was to investigate the efficacy and potential of PCIT in treating aggressive behaviors in conditions other than disruptive, impulse control, and conduct disorders, summarizing the main evidence and identifying factors that influence the effectiveness of the intervention.

## 2. Materials and Methods

### 2.1. Search Strategy

**Figure 1:**
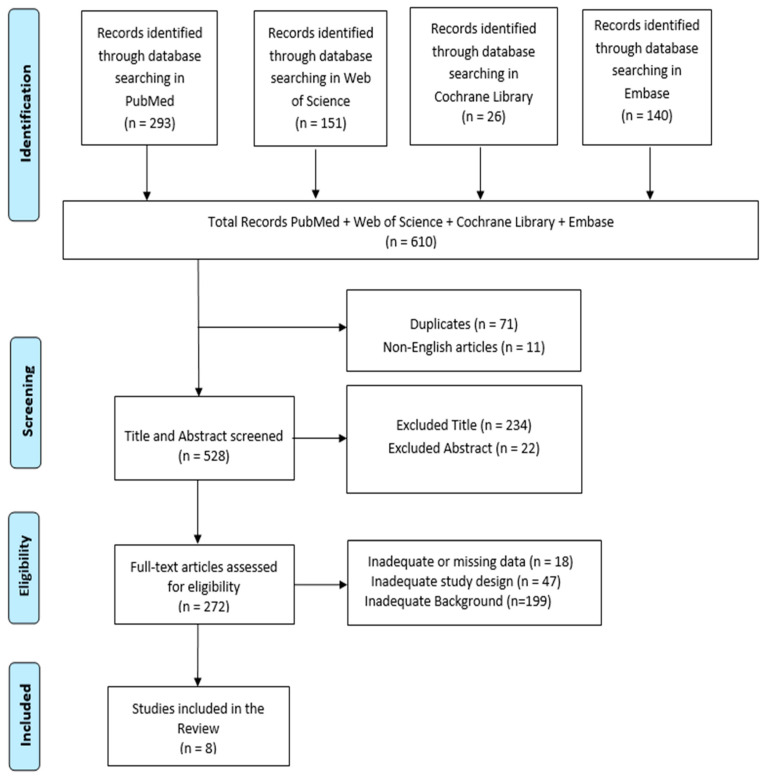
PRISMA 2020 flow diagram of the evaluated studies.

A comprehensive literature search was performed using PubMed, Web of Science, Cochrane Library, and Embase databases, employing the keywords: (All Fields: “Parent–Child Interaction Therapy”) AND (All Fields: “Disruptive Behavior”), with a search period from 2014 to 31 July 2024. The PRISMA (Preferred Reporting Items for Systematic Reviews and Meta-Analyses) flow diagram was utilized to outline the process (identification, screening, eligibility, and inclusion) for selecting relevant studies, as illustrated in Figure 1 [28]. Titles and abstracts from the database searches were independently reviewed. Articles were evaluated for relevance based on predefined inclusion criteria. All titles and abstracts that met these criteria were fully reviewed. Multiple expert teams independently selected articles and analyzed data to minimize bias, discussing discrepancies until consensus was achieved. This review was registered on the Open Science Framework (OSF) managed by the Center for Open Science, Charlottesville, VA, USA. with the following code: TVFQ5.

### 2.2. Eligibility Criteria

Studies that met the following criteria were included: (a) original or protocol studies; (b) articles describing the efficacy of PCIT therapy in reducing disruptive behaviors in populations that fall outside the disruptive, impulse control, and conduct disorders cluster; (c) articles describing the efficacy of PCIT therapy in reducing disruptive behaviors in populations with conditions of Psychosocial Agents of Stress, Emotional and Social Functioning, and Disorders due to Medical and Developmental Conditions; and (d) studies published in English. Articles that did not meet the inclusion criteria were excluded, specifically (a) case reports, letters to the editor, and systematic, integrative, or narrative reviews; (b) studies in populations that fall within the disruptive, impulse control, and conduct disorders cluster; (c) articles that did not describe the efficacy of PCIT therapy in reducing disruptive behaviors in populations with conditions related to psychosocial agents of stress, emotional and social functioning, or disorders due to medical and developmental conditions; (d) reports with overlapping samples; (e) missing data on child developmental outcomes or parenting measures; and (f) articles in languages other than English.

### 2.3. Study Selection and Data Extraction

Two blinded authors (AC, AP) removed duplicates, and the identified references were then screened by title and abstract. Subsequently, full-text articles were evaluated for eligibility based on title, abstract, full-text, and topic specificity. Whenever ratings were discordant, a third investigator (RSC) analyzed the result and reached a consensus. When overlaps were found, the largest study was included. Two authors (AC, AP) blindly analyzed the extracted data independently.

### 2.4. PICO Evaluation

We applied the PICO model (Population, Intervention, Comparison, Outcome) to create our search terms.

The population of interest included children with DBPs across different age groups and demographic backgrounds. The intervention assessed was PCIT, aimed at enhancing parent–child interactions and reducing behavioral problems. The comparison involved PCIT versus other treatments, such as standard behavioral therapy, no treatment, and other therapeutic approaches. The outcome of interest was the reduction in disruptive behaviors. Outcome tools included standardized assessment scales incorporating behavioral observations. This systematic review aggregated data from different populations and settings to give an overall picture of the effectiveness of PCIT in the management of DBPs.

## 3. Results

### 3.1. Synthesis of Evidence

In total, 610 articles were identified. After screening, 71 articles were removed due to duplication, and 11 articles were excluded because they were not published in English. This left 528 articles for title and abstract screening. Finally, 18 articles were excluded because of inadequate or missing data, 47 due to inadequate study design, and 199 due to insufficient background, including studies that lacked relevance, theoretical foundation, or connection to the research aim (Figure 1). Eight research articles met the inclusion criteria and were included in this review. These studies are summarized in Table 1.

The studies discussed in this review explored the effectiveness of PCIT in reducing DBPs across diverse populations and settings. Eight articles explored the versatility and effectiveness of PCIT in treating DBPs [29,30,31,32,33,34,35,36].

In most of the included studies (n = 5), the mean number of sessions performed was 14.8. The other three remaining studies [31,34,35] did not report the exact number of sessions but provided average values: 21 sessions [31], 10 sessions [34], and 10.84 [35]. The overall age range across the included studies was 1 to 10 years. Of the included studies, five studies were randomized controlled trials (RCTs) [29,30,31,32,33], two were observational [34,35], and one was a pilot study [36]. The studies included PCIT interventions for DBP in different populations, including children with DBPs [29,30,31,32,35,36] and children with both DBPs and Autism Spectrum Disorder (ASD) [33,34].

### 3.2. PCIT Intervention in DBP

Six studies included in the review examined the effectiveness of PCIT in treating children with behavioral problems without additional medical conditions or diagnoses [29,30,31,32,35,36]. These studies focused on different populations and settings, evaluating the application of PCIT in high-risk families, customized versions for culturally diverse groups, intensive interventions, and adaptations for toddler children.

In high-risk settings, such as foster families [29] and child welfare programs [35], PCIT has been adapted to address challenges related to child trauma and parenting stress. Studies have shown that the intervention significantly improved the parent–child relationship, reducing externalizing symptoms in children [29] and strengthening parenting practices [29,32]. Parents reported a significant reduction in stress, accompanied by an increase in their coping skills, including improvements in inhibitory control and emotional regulation [32]. These findings highlight how PCIT can be effective even in high-vulnerability settings, promoting positive changes in both children and caregivers and helping parents establish more positive and sensitive parenting practices [29,32].

In addition, a personalized version of PCIT for culturally diverse families using the PersIn framework showed positive changes in both children’s behaviors and parenting practices. The results obtained were comparable to those of standard interventions, showing that personalization can improve adherence without compromising treatment effectiveness [36].

Another significant variant was PCIT-Toddlers (PCIT-T), designed for very young children (aged 12 to 24 months). This version showed improvements in parenting skills and a significant reduction in problem behaviors. It also strengthened secure attachment between parents and children, suggesting that early intervention can prevent future problems and support healthy social-emotional development [35].

The adoption of intensive versions, such as I-PCIT, has been shown to be particularly effective for families with high parental stress. These adaptations have facilitated rapid symptom improvement and reduced treatment dropout rates, demonstrating the flexibility of PCIT and its ability to respond to diverse clinical needs [30].

Finally, a study conducted in real-world clinical settings showed that PCIT outperforms usual treatment (TAU) in improving parenting practices and reducing problem behaviors in children. The treatment benefits were maintained over the long term (up to 18 months after the intervention), confirming the sustainability of the results [31].

### 3.3. PCIT Intervention in ASDd

Two studies [33,34] explored the effectiveness of PCIT for children with ASD and disruptive behaviors, focusing on different contexts (one in clinical settings [33] and the other in community-based settings [34]). Both studies reveal that PCIT significantly reduces disruptive behaviors and improves parent–child relationships.

In the study by Allen et al. [33], PCIT was tested in a clinical trial setting with children diagnosed with ASD and disruptive behaviors. Tshe results demonstrated significant reductions in disruptive behaviors and improved compliance, regardless of the severity of ASD symptoms. Furthermore, the study highlighted improvements in parental stress and mental health as key secondary outcomes. The researchers noted that these behavioral improvements were consistent across children with varying levels of ASD severity and receptive language skills, highlighting the versatility of PCIT in treating children with varying functional levels across the autism spectrum.

In contrast, the second study by Quetsch et al. [34] examined the effectiveness of PCIT when implemented in community-based settings by non-specialist clinicians. This study compared outcomes between children with ASD or developmental delays (DDs) and children without these diagnoses. It found that both groups experienced significant reductions in disruptive behaviors, with no major differences in the number of sessions required or the length of treatment between the groups. This suggests that PCIT, even when administered by non-specialist clinicians, can be highly effective for children with ASD in real-world settings. The study also reported that caregivers perceived similar improvements in the parent–child relationship, and graduation rates from PCIT were consistent between families with and without children with ASD or DD. Both studies highlight the effectiveness of PCIT in reducing disruptive behaviors and improving family dynamics, regardless of ASD severity or clinician skill level. These findings suggest that PCIT is a flexible, evidence-based intervention that can be successfully applied in both controlled and real-world settings, providing meaningful improvements for children with ASD and their families.

## 4. Discussion

This systematic review examined the effectiveness of PCIT in reducing DBPs by analyzing outcomes in different populations and settings. Studies conducted in high-risk maltreatment settings and in foster families demonstrated the effectiveness of PCIT in improving the parent–child relationship and reducing externalizing symptoms in children [29,32]. In particular, the therapy has demonstrated significant benefits in reducing parental stress [29] and enhancing emotional regulation and inhibitory control skills among caregivers [32]. These findings are particularly relevant considering that foster families often face high levels of stress and prior trauma in children [37], and that child maltreatment broadly impacts multiple domains of child development [38]. PCIT appears to be an effective strategy in these situations, successfully improving parenting practices and promoting a more stable and positive family environment.

In the context of neurodevelopmental disorders, PCIT appears to be effective for children with ASD, as evidenced by studies conducted in both clinical [33] and community settings [34]. In one clinical trial, PCIT led to significant reductions in disruptive behaviors in children with ASD, regardless of their support needs [33]. Importantly, interventions also improved caregivers’ stress and mental health, underscoring the benefit of therapy not only for the child but for the entire family unit. Furthermore, in community settings, with non-specialist therapists, the results appear comparable to those in specialized settings, indicating that PCIT can also be implemented effectively in other settings where resources may be limited [34]. These results highlight the flexibility and accessibility of the therapy, which can be adapted to different settings without compromising its effectiveness. PCIT in non-specialized settings could be considered relevant, as it could be one of the desirable solutions to break down significant barriers in accessing essential services and supports. In fact, the complex nature of autism requires a coordinated set of services and supports involving various sectors and providers, adapted to the different stages of an autistic individual’s life [39].

However, individuals with autism and their families often face significant barriers to accessing rehabilitative services, which can compromise the continuity and effectiveness of necessary treatment [40].

From an early intervention perspective, the PCIT-Toddler variant, designed for children aged 12 to 24 months, has been shown to improve parenting skills and significantly reduce problem behaviors [35]. This early adaptation of therapy also strengthened secure attachment between parent and child, suggesting that early interventions can prevent the development of future problems and support healthy social-emotional development. The findings underscore the importance of intervening in the early years of life, when disruptive behaviors are more malleable, and parents are particularly receptive to changes in educational practices. These data align with other studies that consider early parenting interventions to be effective in improving parent–child relationships and supporting early cognitive, language, motor, and socio-emotional development [41,42].

When analyzing the frequency and duration of PCIT, intensive adaptations, such as I-PCIT, were also found to be particularly effective for families with high levels of parental stress, facilitating rapid improvements in symptoms and reducing treatment dropout rates [30]. The efficacy of PCIT was also considered in comparison to usual treatment in children with DBPs. The results show greater improvement with PCIT compared to usual treatment, with benefits maintained up to 18 months after the intervention [31]. In addition, using the PersIn framework to personalize PCIT in culturally diverse contexts showed that therapy can be modified to meet cultural norms, improving treatment adherence and maintaining efficacy. Personalization has allowed the use of communication strategies and disciplinary techniques that are better suited to families’ cultural contexts, fostering a more engaging and productive therapeutic experience [36].

The use of culturally adaptive PCIT seems to be a viable approach, considering the importance of establishing a therapeutic alliance with the family system. It is likely that cultural adaptation, by modifying intervention methods to respect and enhance the value system of the culturally diverse family, can help to avoid techniques that conflict with cultural norms. The use of communication strategies and a disciplinary approach that adapts to families’ cultural contexts could result in increased practices learned during therapy, facilitating the experience and making it more engaging and effective. This is in line with qualitative research conducted by Cardona et al. (2021) that highlights the importance of culturally and contextually adapting parenting interventions [43].

Therefore, the results of the present work emphasize that PCIT is a versatile and adaptable intervention, capable of addressing a wide range of behavioral problems in different settings and populations.

However, this systematic review of PCIT for DBPs includes some notable strengths and important limitations. Its strength lies in the robust analysis based on several studies demonstrating that PCIT is versatile, effective, and easily applicable across different populations and contexts. The application of the PICO model and PRISMA guidelines ensures a rigorous and systematic approach, improving the reliability of the results. The adaptability of PCIT is evident in its successful application across different family systems and cultural contexts. Its effectiveness in community-based settings underscores the breadth of its applicability. This review also describes some outcome measures that include reductions in disruptive behaviors and improvements in parent–child relationships, thus providing a comprehensive view of the benefits of PCIT. However, there are obvious limitations: the review calls for further research on long-term PCIT, which remains under-researched, and variable study designs, sample sizes, and follow-up periods raise issues regarding the comparability of outcomes. Additionally, although the included PCIT studies are limited, with only nine being analyzed, its use with children with co-occurring conditions was reviewed. This also highlights the inadequacy of addressing such co-occurring conditions in a sufficiently comprehensive manner. Most studies emphasize short-term outcomes, overlooking a major deficit in the long-term sustainability of effects. At a minimum, the efficacy demonstrated in controlled research settings may not translate as well into real-world applications, particularly in areas with little or no access to specialized therapy. Although this review affirms the efficacy and flexibility of PCIT, it also points to areas that need further examination to improve its generalizability and long-term impact.

## 5. Conclusions and Future Directions

This systematic review provides an overview of PCIT for DBPs, highlighting its general efficacy and adaptability across settings and populations. In terms of therapeutic efficacy, substantial success has been reported with PCIT in reducing disruptive behaviors, improving the quality of parent–child relationships, and reducing parental stress. Innovations such as PCIT-CU for children with CU traits and MY PCIT for culturally tailored interventions have further expanded its reach and effectiveness. Evidence supporting its application in specialized and community-based settings speaks to its strength and cost-effectiveness. However, these promising findings have opened up future areas of research. One important area that needs to be explored is the long-term effectiveness of PCIT. In this regard, more studies are needed to better understand the effects of the intervention over longer periods. Further research on its adaptability in multicultural settings and its effectiveness in comorbid conditions is also indicated. For example, the use of technology to enhance PCIT or web-based applications could be one such future avenue to increase its uptake and effectiveness. Future research should focus on refining PCIT to target specific behavioral problems, such as primary and secondary CU traits, and optimizing its delivery in different settings. Further evaluation of the adaptability of PCIT, including integrating technology and culturally sensitive practices, will remain critical to enhance its future role in effectively treating children with DBPs and promoting the overall well-being of the child and family.

## Figures and Tables

**Table 1:** Sample characteristics and design of selected studies.

Author	Aim	Study Design/Intervention	Treatment Period	Sample Size (N. Children; N. Parents)	M:F	Children Years Range (M; SD)	Outcome Measures	Main Findings	Study Limitations	Statistical Analyses
Mersky et al. 2016 [29]	Evaluating the effectiveness of a novel PCIT model tailored for foster families, specifically examining the differential effects of two intervention doses on children’s externalizing and internalizing symptoms	Randomized Controlled Trial	After completing 2 days of training and 8 weeks of home care, the intervention ended for the short PCIT groups. The extended PCIT groups continued to participate in an additional training session, with 6 more weeks of homework and telephone consultations.	102 children NR parents	0.85:1	3–6 (M 4.6; SD NR)	CBCL, ECBI	PCIT, both short and extended, improved externalizing and internalizing symptoms compared to the control group. Extended PCIT showed sustained improvements after 8 weeks, whereas short PCIT slowed over time.	The study’s limitations include potential bias from non-blinded parent raters, a brief observation period restricting long-term assessment, and a small sample size limiting generalizability, especially to stable, nonrelative foster placements.	Changes over time were analyzed using a mixed-model repeated measures analysis of covariance.
Graziano et al. 2020 [30]	To compare the effectiveness of a more intensive and brief version of PCIT (I-PCT) with a limited-duration PCIT format	Randomized Controlled Trial	I-PCIT: 60 to 90 min sessions, 5 days a week for two weeks PCIT: 60 to 90 min sessions once a week for 10 weeks	60 children NR parents	1.86:1	2–7 (M 4.33; SD 1.29)	ECBI, DPICS, PSI-SF, PS, TAI	Both groups showed reduced externalizing behaviors (ECBI), improved parenting skills (DPICS), and decreased parenting stress (PSI-SF), with gains maintained at follow-up. Weekly PCIT reported slightly greater reductions in externalizing problems and “Laxness” than I-PCIT. Both groups were highly satisfied with treatment.	The study’s limitations include an ethnically homogeneous sample (85% Latinx), a short follow-up period limiting long-term insights, and no data on children’s school behavior to assess treatment generalizability.	-Non-Inferiority Test -Multivariate Repeated Measures NOVA -Follow-Up Contrasts with Bonferroni Correction -Cohen’s d Effect Calculation -Moderation Analysis
Bjorset et al. 2016 [31]	To compare the outcomes of PCIT versus TAU in children with behavior problems	Randomized Controlled Trial	PCIT Treatment Length: on average, families attended 21.14 sessions (SD = 12.04). TAU Treatment Length: the mean number of sessions for the TAU group was 18.84 sessions (SD = 11.78).	81 children NR parents	1.79:1	2–7 (M 5.8; SD NR)	ECBI, CBCL, DPICS	Children receiving PCIT showed greater improvements in behavior problems compared to those receiving TAU. The ECBI and CBCL scores improved more in children in the PCIT group. Parents in the PCIT group also demonstrated significant improvements (DPICS).	Lack of a post-treatment diagnostic assessment and the absence of formal treatment fidelity measures. Additionally, the therapy environment with co-therapists may not reflect regular clinical practice, and the attrition rates pose concerns for generalizability.	Linear growth curve analyses were used to evaluate the data.
Showrn et al. 2024 [32]	To improve positive parenting skills, reduce negative parenting behaviors, and support self-regulation skills of parents of children at risk of maltreatment	Randomized Controlled Trial	PCIT intervention condition: 9 CDI sessions (one skill teaching, eight coaching) and 11 PDI sessions (one skill teaching, 10 coaching).	204 children 204 parents	1.21:1 NS	3–7 (M 4.8 SD 1.4) 18-64 (M 32.3 SD 6.4)	BRIEF-A, SSRT, SASB, DPICS, ECBI	The PCIT group showed fewer emotional control problems (BRIEF-A), lower SSRT scores, and greater increases in SASB Self-Affirming scores compared to the control group. No differences were found in parental commands (DPICS), but children in the PCIT group had lower ECBI behavior problem scores.	The study lacks follow-up, has limited generalizability, minimal treatment involvement measures, and overlooks parental comorbidities.	-ITT analysis -Similar multiple regression models -Mediation and Moderation in Repeated measurement designs
Allen et al. 2022 [33]	To evaluate the effectiveness of PCIT in reducing disruptive behaviors among ASD children without intellectual disabilities	Randomized Controlled Trial	14sessions between 60 and 90 min on a weekly basis	55 children 55 mothers 8 fathers	5.88:1	4–10 (M 7.15; SD 1.72)	BASC-2, ECBI, PSI-SF, PLOC-SF, BDI-II, TAI, DPICS	The treatment reduced behavior problems (ECBI, BASC) and parenting stress (PSI-SF) in the experimental group, with parents finding the techniques useful (TAI) and improving parenting skills (DPICS). No differences were noted in PLOC-SF and BDI-II.	The study lacked an alternative treatment control, making the results provisional. Further analysis of the effectiveness of treatment across support is needed.	Comparative analyses were conducted between the PCIT and control groups using repeated measures MANOVA to assess treatment effects.
Quetsch et al. 2024 [34]	To examine the effectiveness of PCIT in reducing disruptive behaviors and improving parent–child relationships among children with and without ASD/DD in community-based settings	Comparative Study	The families participated in a number of sessions that varied from 9 to 11 sessions. The treatment lasted approximately 4 months and one week.	Parents 2435	4:1	3–7 (M 5.06; SD 1.51)	ECBI	PCIT led to significant reductions in disruptive behaviors and improvements in the parent–child relationship for both children with ASD/DD and those without.	The study’s limitations include non-specialized clinician training, potential underreporting of ASD diagnoses, missing data from routine collection, and limited generalizability to diverse populations or settings.	Mixed ANOVA, Chi-square, repeated measures ANOVA
Kohlhoff et al. 2020 [35]	To evaluate the results of the first phase of the PCIT-T in reducing children’s problem behaviour, parenting skills, parental stress and maternal depression	Longitudinal Observational Study	8 weeks; the mean number of PCIT-T sessions was 10.84.	56 children 56 mothers 66 children 66 mothers	5:3	1–2 (M 1.58; SD 0.20) 19–43(M 31.89 SD 4.79)	DPIC IV, EAS, CBCL, PSI-SF, EPDS, SSP	Parenting behaviors (DPICS-IV, EAS) and child behavior problems improved significantly at Time 2 and Time 3. Parenting stress improvements (PSI-SF) persisted at follow-up, whereas maternal depressive symptoms (EDPS) did not. Over 70% of children transitioned to secure attachment and 85% to organized patterns (SSP).	They did not use a randomized controlled design, limiting the ability to establish causality. The sample size was relatively small, and the study was conducted in a single community-based clinic in Australia, which may limit the generalizability of the findings.	Linear Mixed, Models for repeated measures, Generalized Linear Mixed Models with binomial distribution, Reliable Change Index, Cohen’s d Effect Sizes, Independent sample *t*-tests
Yeh et al. 2022 [36]	Evaluate the feasibility and effectiveness of a culturally tailored adaptation of PCIT (MY PCIT).	Pilot Study	14 sessions	32 children 32 parents	1.91:1 15:1	2–7 (M 4.66; SD 1.33) (M 37.94; SD 8.25)	ECBI, DPICS-IV, PSI-4 SF, CBCL	Treatment led to significant reductions in child behavior problems (ECBI, CBCL) and increases in child compliance (53% to 65%). Parenting behaviors improved with more “doing skills” (+16) and fewer “don’t-doing skills” (−13) (DPICS-IV). Parenting stress (PSI) also decreased significantly across all subscales.	The small sample size and the absence of a control group reduced the strength of the findings. Cultural and linguistic diversity in the sample was limited, potentially excluding families needing greater treatment personalization.	Statistical tests to analyze pre-to post-treatment data.

Legend: Male (M), Female (F), Not reported (NR), Parent–Child Interaction Therapy (PCIT), Eyberg Child Behavior Inventory (ECBI), Treatment as usual (TAU), Autism spectrum disorder (ASD), Developmental delays (DD), Child Behavior Checklist (CBCL), Dyadic Parent–Child Interaction Coding System (DPICS), Behavior Assessment Scale for Children Second Edition (BASC-2), Parent Stress Index Short Form (PSI-SF), Parenting Locus of Control Short Form (PLOC-SF), Beck Depression Inventory (BDI-II), Therapy Attitude Inventory (TAI), Parent–Child Interaction Therapy with Toddlers (PCIT-T), Dyadic parent–child interaction coding system fourth edition (DPICS-IV), Emotional availability scales (EAS), and Child behavior checklist 1.5–5 (CBCL/1.5–5).

## Data Availability

The data that support the findings of this study are not openly available due to sensitivity concerns but are available from the corresponding author upon reasonable request.
